# Neutrophil-to-lymphocyte ratio and chemotherapy response score as prognostic markers in ovarian cancer patients treated with neoadjuvant chemotherapy

**DOI:** 10.1186/s13048-021-00902-0

**Published:** 2021-11-01

**Authors:** M. Liontos, A. Andrikopoulou, K. Koutsoukos, C. Markellos, E. Skafida, O. Fiste, M. Kaparelou, N. Thomakos, D. Haidopoulos, A. Rodolakis, M. A. Dimopoulos, F. Zagouri

**Affiliations:** 1 Department of Clinical Therapeutics, Alexandra General Hospital, School of Medicine, National and Kapodistrian University of Athens, Athens, Greece; 2 Department of Obstetrics and Gynecology, Alexandra General Hospital, School of Medicine, National and Kapodistrian University of Athens, Athens, Greece

## Abstract

**Background:**

Neoadjuvant chemotherapy (NACT) followed by interval debulking surgery (IDS) is the recommended approach in patients with advanced epithelial ovarian cancer (EOC). However, most patients eventually relapse despite the initial high response rate to chemotherapy. Neutrophil-to-lymphocyte ratio is a well-known biomarker that reflects severe inflammation, critical illness, and mortality in various diseases. Chemotherapy response score (CRS) and neutrophil-to-lymphocyte ratio (NLR) have been identified as potential biomarkers of platinum resistance and disease prognosis. We retrospectively evaluated 132 patients with stage IIIc or IV ovarian/fallopian tube/primary peritoneal cancer who had received NACT followed by IDS from 01/01/2003 to 31/12/2018. CRS was assessed on omental specimens collected from IDS according to ICCR guidelines.

**Results:**

Median age was 64.57 years (SD: 9.72; range 39.2–87.1). Most ovarian tumors were serous epithelial (90.9%; 120/132). An elevated NLR (defined as > 3) was observed in 72% (95/132) of patients in contrast with 28% (37/132) of patients characterized by low NLR status. Median PFS (mPFS) and median overall survival (mOS) were 13.05 months (95% CI: 11.42–14.67)) and 34.69 months (95% CI: 23.26–46.12) respectively. In univariate analysis, CRS3 score was significantly associated with prolonged mPFS (CRS1/2: 12.79 months vs CRS3: 17.7 months; *P* = 0.008). CRS score was not associated with mOS (*P = 0.876*). High NLR was not significantly associated with mPFS (*P = 0.128*), however it was significantly associated with poor mOS (*P = 0.012*). In multivariate analysis, only performance of surgery maintained its statistical significance with both PFS (*P = 0.001*) and OS (*P = 0.008*).

**Conclusion:**

NLR could serve as a useful predictor of OS but not PFS in ovarian cancer patients receiving NACT. In accordance with our previous study, CRS score at omentum was found to be associated with PFS but not OS in ovarian cancer patients treated with NACT and IDS.

## Research highlights

Biomarkers that would predict response to neoadjuvant chemotherapy in advanced ovarian cancer patients are eagerly needed:

• Neutrophil to Lymphocyte Ratio (NLR) is an indicator of systemic inflammatory response to the malignancy.

• NLR was evaluated in 132 patients undergoing Neoadjuvant Chemotherapy for advanced ovarian cancer.

• Elevated NLR was associated with worse prognosis.

• No association between NLR and response to chemotherapy was noted.

## Background

Ovarian cancer is the eighth most common cause of cancer-related mortality in women globally and the leading cause of gynecological cancer-associated mortality in developed countries [1]. It is estimated that in 2020, about 308,069 new cases of ovarian cancer will be diagnosed and approximately 193,811 deaths will be reported worldwide [2]. Epithelial ovarian cancer (EOC) accounts for the 90% of ovarian cancers with high-grade serous ovarian carcinoma consisting the main histological subtype [3]. Despite the progress of cancer treatment during the last decade, over than two thirds of EOC cases are diagnosed at an advanced stage resulting in a 5-year survival rate of only 25% in stage III and IV disease [4]. The recommended approach for advanced-stage EOC is primary debulking surgery (PDS) followed by platinum-based chemotherapy. However, neoadjuvant chemotherapy (NACT) followed by interval debulking surgery (IDS) proved to be non-inferior to primary debulking surgery in stage III/IV patients unable to undergo optimal cytoreduction during PDS [5–9]. NACT approach consists of three or four cycles of upfront chemotherapy followed by IDS with the goal of complete resection. Despite the initial response to chemotherapy (~ 85%), most patients eventually relapse [10]. Therefore, there is an emerging need for markers and prognostic factors evaluating the response to treatment.

Chemotherapy response score (CRS) is a three-tier histopathological scoring system for validating response to NACT [11]. Patients that achieve CRS3 after NACT have improved progression free survival (PFS) and Overall Survival (OS) according to several retrospective studies and their metanalysis [12–23]. On that basis, the International Collaboration on Cancer Reporting (ICCR) incorporated the use of the CRS system in the current ovarian cancer histopathology reporting guide [13]. It would be of interest though to identify biomarkers that could predict which patients will or will not achieve CRS3 after NACT. That would allow patient selection through clinical trials for alternative therapeutic approaches aiming at improving dismal prognosis of these patients.

Recently, our group has shown that lymphocytic infiltration in pre-chemotherapy biopsies is associated with CRS3 after NACT. This indicates that response to chemotherapy could be associated with the immunological response and the inflammatory milieu of the tumor. Neutrophil-to-lymphocyte ratio (NLR) that serves as a measure of systemic inflammatory response is a prognostic biomarker that has been widely explored in oncology. NLR is easily measured through a complete blood count and could be determined under most circumstances. In ovarian cancer patients, there is evidence supporting preoperative NLR clinical utility not only in outcome prediction [24–32] but also in distinguishing benign from malignant ovarian tumors [33–35]. NLR has been associated with CD4+ helper T cells (Th17) count and IL-8 and IL-6 plasma concentration [36, 37], levels of stromal IL-2 [38] and serum fibrinogen [39]. Overall, higher NLR values positively correlated with platinum resistance and poor prognosis.

In this context, we evaluated the predictive and prognostic role of NLR in advanced ovarian cancer patients treated with NACT followed by IDS and examined its association with CRS.

## Methods

We retrospectively identified patients with stage IIIc or IV ovarian/fallopian tube/primary peritoneal cancer who had received NACT followed by IDS during a 15-year period (January 2003–December 2018) in our institutional database. Patients with coexisting hematological malignancies, systemic administration of corticosteroids or any inflammatory conditions that could affect blood counts were excluded. Our institution has been certified by the European Society of Gyenocologic Oncology (ESGO) as a center of excellence for the treatment of ovarian cancer. The study has been performed in accordance with the 1964 Helsinki Declaration and has been approved by the Institutional Review Board of Alexandra University Hospital (Protocol Number: 513/15-07-2020). Patients were selected for NACT and IDS if it was judged by the experienced Gynecologic Oncologists that they could not be debulked upfront to no residual tumor. Assessment involved imaging studies and/or laparoscopic evaluation. At least 3 cycles of NACT and adequate pathology specimens were required for inclusion to the analysis. IDS was performed at 4–6 weeks from last dose of chemotherapy. Since 2013, that bevacizumab administration in the frontline setting became the standard of care in the country, all patients were planned to receive bevacizumab post IDS, unless contraindicated. All women had provided informed consent for their treatment as well as for their use of medical records for research purposes. Clinicopathological characteristics including age at diagnosis, stage and histology of the disease, CA125 at diagnosis, residual disease at IDS, BRCA mutation status, type of chemotherapy administered, neutrophil and lymphocyte count prior to neoadjuvant chemotherapy, progression of the disease and overall survival were collected from the medical records of the patients. Optimal debulking was defined as maximum residual tumor < 1 cm in diameter after IDS.

### Pathology review

Tissue slides collected from IDS were thoroughly reviewed by an experienced gynecologic pathologist trained in assessment of Bohm’s score (MS). CRS was assessed on omental specimens according to ICCR guidelines [13].

### Neutrophil to lymphocyte ratio evaluation

Complete blood cell counts (CBC) were collected from all patients prior to commencing chemotherapy. NLR was defined as the absolute neutrophil count divided by the absolute lymphocyte count. An elevated NLR was defined as an NLR > 3 based on previous studies [40–44].

### Statistical analysis

Continuous variables were summarized with the use of descriptive statistical measures [median and percentiles (25th,75th)] and categorical variables were displayed as frequency tables (N, %). The outcome of the debulking surgery was classified as complete (no residual disease) or optimal (residual disease below 1 cm). Overall Survival (OS) was defined as the time between the start of chemotherapy and the date of death from any cause. Progression-free Survival (PFS) was defined as the time between the start of chemotherapy and the date of progression. Alive patients were censored at the date of last contact. Kaplan-Meier estimates were used to describe and visualize the effect of categorical variables on OS and PFS. Log-rank tests were used to explore the prognostic value of categorical variables on clinical outcomes. The association of these factors with OS was assessed through HRs and their 95% confidence intervals estimated from univariate Cox proportional hazards models. Interactions between covariates and time varying effects were studied. Multivariate Cox-regression model of CRS omentum adjusted for age, Stage, and Debulking surgery was presented. Proportional hazards (PH) assumption was graphically assessed using plots of -ln{−ln(survival)} curves for each category of the covariates versus ln (analysis time). All statistical analyses were performed using the STATA/SE 16.0 software (Copyright 1985–2019 StataCorp LP).

## Results

### Patient baseline characteristics

Overall, 132 ovarian cancer patients treated with NACT were identified. Demographic, clinical and pathologic characteristics are presented in Table 1*.* In brief, median age was 64.57 years (SD: 9.72; range 39.2–87.1). Most ovarian tumors were serous epithelial (90.9%; 120/132), while there were isolated cases of endometrioid (0.8%; 1/132), poorly differentiated (1.5%; 2/132) tumors and nine cases (6.8%) where histological subtype was not specified. Ninety patients (68.2%) had stage IIIC disease and 41 patients (31.1%) had stage IV disease.

**Table 1:** Baseline characteristics

Characteristics	No. of patients (%)
**Age (yr)**	
**Median**	64.57
**Range**	39.2–87.1
**Histological type**	
**HGSC**	120 (90.9%)
**Endometrioid**	1 (0.8%)
**Undifferentiated**	2 (1.5%)
**Not specified**	9 (6.8%)
**FIGO stage**	
**IIIC**	90 (68.2%)
**IV**	41 (31.1%)
**Not specified**	1 (0.8%)
**Regimen of NAC**	
**Taxane+carboplatin**	113 (85.6%)
**Other**	16 (12.1%)
**Not specified**	3 (2.3%)
**Debulking surgery**	
**Yes**	103 (78%)
**No**	29 (22%)
**ECOG score**	
**0–1**	93 (70.4%)
**2–3**	25 (18.9%)
**4**	2 (1.5%)
**Not specified**	12 (9.1%)
**NLR**	
**≤ 3**	37 (28%)
**> 3**	95 (72%)
**CRS**	
**CRS1/2**	50 (68.5%)
**CRS3**	23 (31.5%)
**Debulking**	
**Optimal**	60 (58.2%)
**Suboptimal**	33 (32%)
**Not specified**	10 (9.7%)
**BRCA testing**	
**Yes**	46 (34.8%)
**No**	85 (64.4%)
**Not specified**	1 (0.8%)
**BRCA mutation**	
**Yes**	11 (8.3%)
**No**	36 (27.3%)
**Unknown**	85 (64.4%)
**Bevacizumab treatment**	
**Yes**	42 (31.8%)
**No**	74 (56.1%)
**Not specified**	16 (12.1%)

A significant percentage of patients had performance status (PS) ≤1 in our cohort (ECOG 0: 38.6%; 51/132 and ECOG 1: 31.8%; 42/132 respectively). Overall, 78% (103/132) of patients treated with three or four cycles of NACT eventually underwent IDS. The remaining 29 patients (22%) never received a debulking cytoreductive surgery either due to rapid clinical deterioration and death prior to surgery, failure to achieve a disease regression to a surgically resectable extent or due to patients’ preference. Debulking was optimal in 58.2% (60/103) of the cases and suboptimal in 32% (33/103). Initial chemotherapy consisted of paclitaxel/carboplatin in the majority of cases (85.6%; 113/132) while the remaining received carboplatin monotherapy. Testing for BRCA1/2 mutations was conducted in 34.8% of cases (46/132). BRCA mutations were detected in 11 cases (8.3%). Bevacizumab was administered in 31.8% (42/132) of patients either as first line maintenance treatment or as a subsequent line.

CRS score was available in 73 cases. 19 (26%), 31(42.5%) and 23 (31.5%) patients had ovarian CRS of 1, 2 and 3 respectively. Patients were classified in two distinct groups: one group with CRS1/2 (68.5%; 50/73) versus those with CRS3 (31.5%; 23/73).

### NLR and its assocations with clinical characteristics in ovarian cancer

Median value of neutrophil-to-lymphocyte ratio (NLR) was 4.18 (SD: 3.38; range 1.1–23.8). An elevated NLR (defined as > 3) was observed in 72% (95/132) of patients in contrast with 28% (37/132) of patients characterized by low NLR status. Attempting to dissect the population of patients with high NLR, we evaluated the association of NLR with clinicopathological factors. High NLR was significantly associated with poor performance status (ECOG-PS ≥ 2) (*P =* 0.001), but not stage (*P =* 1.000) or age (*P =* 0.441). In relation to the applied treatments, high NLR was not associated with monotherapy carboplatin as initial chemotherapy (*P =* 0.380) but was statistically significantly correlated with inability to perform debulking surgery (*P =* 0.019), and omittion of bevacizumab as a maintenance treatment (*P =* 0.004). For the 73 cases that CRS data were available, no statistical significant association with NLR was shown (*P =* 0.321).

### Survival analysis

**Fig. 1:**
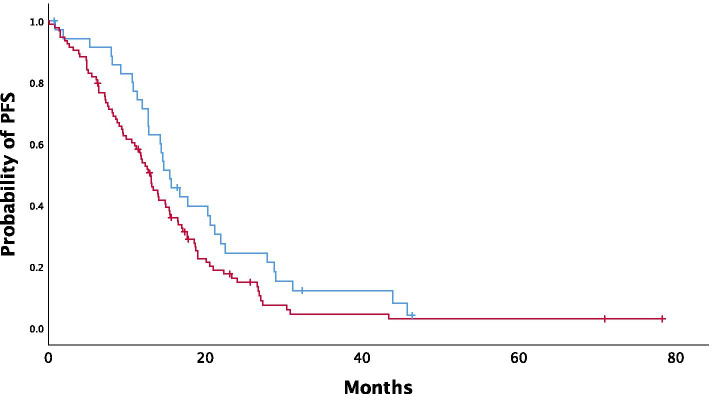
Kaplan-Meier curves depicting progression-free survival (PFS) according to NLR status (blue line:NLR-low, red line: NLR-high)

**Fig. 2:**
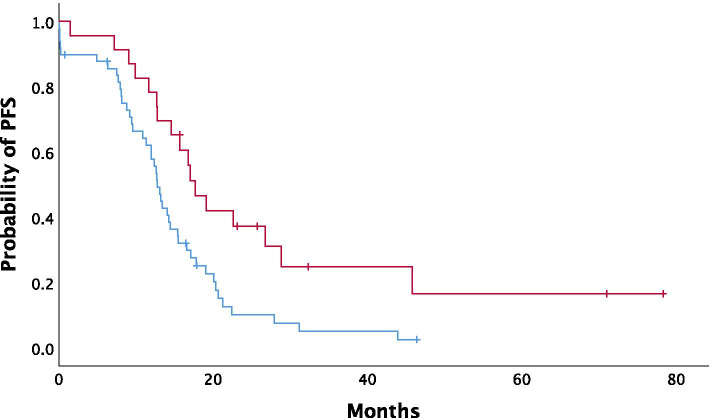
Kaplan-Meier curves depicting progression-free survival (PFS) according to CRS (blue line: CRS1/2, red line: CRS3)

Overall, median PFS (mPFS) was 13.05 months (95% CI: 11.42–14.67). In univariate analysis, NLR was not significantly associated with mPFS (*P = 0.128*) (Fig. 1). mPFS was 14.56 months in patients with low NLR (NLR < 3) (95% CI: 11.44–17.67) in contrast to 12.39 months (95% CI: 10.64–14.13) in the high NLR population. In contrast, CRS3 score was significantly associated with prolonged mPFS (CRS1/2: 12.79 months vs CRS3: 17.7 months; *P* = 0.008), along with other clinical parameters and treatment characteristics such as performance of cytoreductive surgery (*P* < 0.001), bevacizumab administration (*P* = 0.02), ECOG performance status < 2 (*P =* 0.061) and presence of BRCA1/2 mutations (*P* = 0.003) (Fig. 2).

**Fig. 3:**
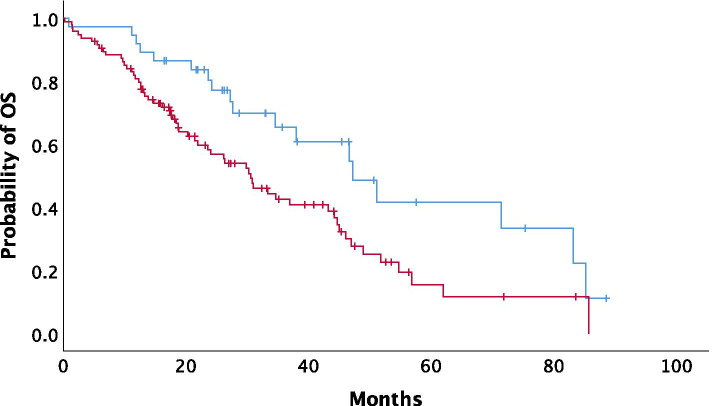
Association between OS and NLR status. (blue line: NLR-low, red line: NLR-high)

In the total population, median overall survival (mOS) was 34.69 months (95% CI: 23.26–46.12). High NLR status was significantly associated with poor mOS (*P = 0.012*). Median OS was 47.18 months for patients with low NLR values before chemotherapy initiation (95% CI: 32.77–61.59; SD: 7.35) versus 30.59 months in patients with high NLR (95% CI: 23.36–37.82; SD: 3.68) (Fig. 3*)*.

**Fig. 4:**
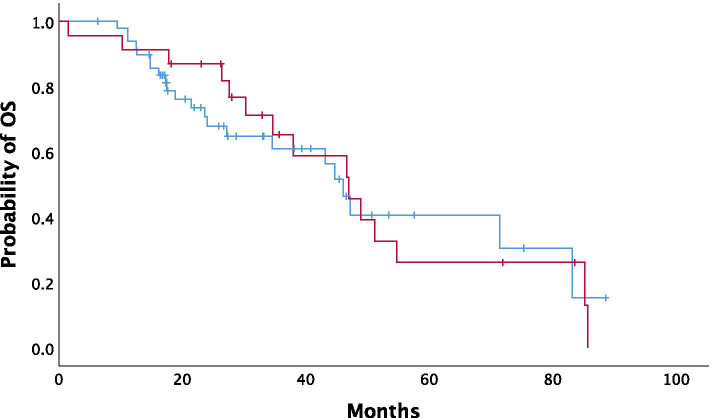
Association of chemotherapy response score (CRS) with overall survival. (blue line: CRS1/2, red line: CRS3)

In univariate analysis, CRS score was not associated with mOS (*P = 0.876*) (Fig. 4). Performance status (ECOG ≤1) (*P* < 0.001), bevacizumab administration (*P* < 0.001) and performance of cytoreductive surgery (*P* < 0.001) demonstrated a strong correlation with mOS. In contrast, age over 65 (*P = 0.129*) and quality of debulking (*P = 0.568*) was not significantly associated with mOS.

On multivariate analysis, the performance of surgery was the single factor statistically significantly associated with both PFS (*P = 0.001*) (Table 2) and OS (*P = 0.008*) (Table 3). NLR failed to maintain its statistical significance on multivariate analysis.

**Table 2:** Multivariate analysis for PFS

	***P*** Value	95% CI	
		Lower	Upper
**Bevacizumab administration**	.275	.466	1.243
**ECOG score**	.323	.355	1.406
**Surgery**	**.001**	1.704	6.973
**Age > 65**	.413	.769	1.893
**NLR**	.903	.618	1.724
**FIGO stage**	.229	.836	1.044

**Table 3:** Multivariate analysis for OS

	***P*** Value	95% CI	
		Lower	Upper
**Bevacizumab administration**	.134	.309	1.169
**ECOG score**	.271	719	3.231
**Surgery**	.**008**	1.287	5.201
**Age > 65**	.732	.632	1.921
**NLR**	.569	.632	2.302
**FIGO stage**	.784	.859	1.122

## Discussion

We conducted this retrospective study to evaluate the role of CRS and NLR as independent biomarkers in advanced ovarian cancer patients treated with NACT and IDS. On univariate analysis, NLR (high NLR > 3 vs low NLR ≤ 3) was significantly associated with OS *(P = 0.012).* In addition, NLR inversely correlated with mPFS, although the association was not statistically significant (*P = 0.128).* PFS was 14.56 months in patients with low NLR values prior to chemotherapy initiation and 12.39 months in those with high NLR values. Although CRS3 score was significantly associated with prolonged mPFS (CRS1/2: 12.79 months vs CRS3: 17.7 months; *P* = 0.008), this correlation was not confirmed with mOS (*P = 0.876*). Neither of these biomarkers was significantly associated with PFS or OS on multivariate analysis.

Systemic inflammation caused by malignancies is often expressed as neutrophilia, thrombocytosis and relative lymphocytopenia in the peripheral blood [45, 46]. Neutrophils contribute to the development of an inflammatory microenvironment, promoting tumor growth, vascularization and metastasis. In addition, neutrophils attenuate the immune system by suppressing CD8+ T lymphocyte antitumor response through the release of nitric oxide synthase (iNOS) or arginase 1 (ARG1) [47]. Neutrophils facilitate tumor initiation and proliferation by producing MMP9 and transferring neutrophil elastase to cancer cells. In ovarian cancer patients, neutrophils possess intensified functions such as increased reactive oxygen species (ROS) production and increased adhesive ability [48] and may be implicated in disease progression by spreading to the omentum as early as before metastasis becomes detectable [49]. Neutrophils become abundant in the omentum and facilitate ovarian cancer cell implantation. Simultaneously, lower counts of lymphocytes may be related to the existence of a primary immunodeficiency. Lymphocytopenia may imply a poor lymphocyte-mediated immune response to malignancy and therefore an increased possibility of tumor recurrence. In this context, NLR was used as a representative index of inflammation and was evaluated in ovarian cancer. Interestingly, NLR has been proposed as useful marker for the preoperative discrimination of ovarian masses [50–52]. High NLR ratio could serve as an accurate discriminator of malignant ovarian masses in the preoperative setting.

In ovarian cancer, the prognostic significance of NLR has been shown in several studies [26, 42, 44, 53] including metanalyses encompassing studies in either gynecological cancers or exclusively ovarian carcinomas [54]. In all the above referenced studies, increased NLR values were associated with worse prognosis and resistance to platinum-based therapy. It should be noted though that different cut-off limits were evaluated in these studies that enrolled mainly patients treated with primary debulking surgery.

Our study enrolled solely patients that underwent IDS after receiving three cycles of NACT. We report that NLR is associated with OS, but not PFS on univariate analysis. There is only a limited number of studies addressing this specific population. *Kim* et al. were the first to evaluate the predictive value of pretreatment NLR in ovarian cancer patients undergoing neoadjuvant chemotherapy in an Asian population [43]. In accordance with our findings, an elevated NLR defined as over 3.81 was independently associated with worse OS, but not PFS [43]. Of note, there was a significant association between PFS and the dynamic change in NLR during neoadjuvant chemotherapy (HR, 2.07; 95% CI, 1.32–3.25). A similar study used a cut-off value of 6 and again increased NLR was significantly associated with worse OS in univariate analysis (HR 1.06, 95% CI 1.01–1.13, *P* = 0.040) but not in multivariate analysis (HR 1.05, 95% CI 0.99–1.12, *P* = 0.080) [26]. There was also no significant association between NLR and PFS in this population (*P* = 0.084). Finally, a sub analysis of MITO 24 study aimed to evaluate if an elevated NLR (defined as NLR > 3 alike our study) could associate with clinical outcome [42]. The study included both patients treated with PDS and patients undergoing NACT and IDS approach. Elevated NLR was independently associated with PFS (HR = 1.23, 95% CI 1.10–1.37), OS (HR = 1.41, 95% CI 1.23–1.62) and PFI at 6 months (OR = 2.52, 95% CI 1.30–4.87, *p* = 0.006) and 12 months (OR = 2.05, 95% CI 1.05–4.01, *p* = 0.036) [42]. Interestingly, NLR lost its predictive value within the bevacizumab-treated population. In our study we have included both patients that received bevacizumab and patients that did not. Bevacizumab administration was significantly associated with PFS and OS, but this association lost its significance in the multivariate analysis indicating that bevacizumab administration is not an independent determinant of survival in these patients. In addition, the groups of patients that received or not bevacizumab in our study were heterogenous and further evaluation of NLR predictive value within each one lacks clinical significance. Overall, our results are in agreement with those observed in other studies addressing women treated with NACT and IDS.

Apart from NLR, other systemic inflammatory response markers have gained attention during the past years. Platelet-to-lymphocyte ratio (PLR) has been proposed as an indicator of prognosis in epithelial ovarian cancer in numerous studies [55, 56]. Fibrinogen, as a key factor of coagulation cascade, also implicates solid tumors and cancer aggressiveness. *Yang* et al recently proposed the combination of preoperative fibrinogen and NLR (F-NLR) as a novel prognostic index in ovarian cancer although not in patients treated with IDS [41]. The F-NLR showed greater sensitivity and specificity than did fibrinogen or NLR alone. C-reactive protein (CRP) has been validated as a predictive factor in solid tumors [57]. Moreover, lymphocyte to monocyte ratio has been related with ovarian cancer aggressiveness and clinical outcome in a number of studies [58, 59]. Collectively, NLR could be combined with other systemic markers to increase the accuracy achieved.

Apart though from its prognostic significance, NLR would be of value in ovarian cancer patients undergoing NACT, if it could predict response to chemotherapy as assessed currently by CRS. Bohm et al. initially demonstrated the prognostic significance of CRS score in terms of PFS and platinum sensitivity [11]. This initial observation was followed by others including our work validating the prognostic and/or predictive value of CRS in ovarian cancer patients undergoing NACT [15, 18, 20, 60, 61]. Recently, a metanalysis of published studies have shown that optimal response to NACT - designated as CRS3 – is associated with both FPS and OS benefit and this could be determined by the molecular biology of the disease as CRS3 was more frequently encountered among BRCA1/2 mutant patients [23]. However, this is not supported by other studies [62, 63] while other parameters as is the extent of the disease at initial diagnosis should be taken into consideration when evaluating response to chemotherapy [22, 64, 65]. In our study, no specific association between CRS and NLR at diagnosis was noted. Analogous was the result in the previous study conducted in the Asian population [43]. The number of patients could be a limiting factor for both studies. However, it should be taken into consideration whether this negative finding could really reflect the lack of a biological association. In our previous analysis, increased lymphocytic infiltration was correlated to CRS denoting that the inflammatory milieau of the tumor could determine sensitivity to chemotherapy [19]. This observation was in accordance with the prognostic role of tumor-infiltrating lymphocytes in ovarian cancer [66] corresponding probably to NLR-low tumors. Therefore, larger studies are warranted to fully evaluate this association.

Finally, it should be noted that there is a great diversity regarding the cut-off values of NLR among studies [40]. This raises the question of the biological significance between NLR-high and low populations. Median cut-off for high NLR was 2.95 among studies evaluating OS and 2.79 among studies evaluating PFS in correlation with NLR [40]. NLR threshold applied ranged between 0.89 to 5.03 across different studies. On that basis, we used 3 as the cut-off value and defined NLR > 3 as high NLR value in our understudy population. However, it is evident that 72% of patients in our study were characterized as NLR-high by this binary classification. Obviously, these patients could not represent exclusively a population with adverse prognostic features. More likely, these patients designated as NLR-low in our study correspond to a population with minimal systemic inflammation and better prognostic features. This is most probably the reason that NLR lost its prognostic significance in the multivariate analysis.

Our study has some limitations. Firstly, the data was retrospectively collected and comprise a relatively small patient cohort from a single center. Moreover, there is a bias against ovarian cancer patients that could not eventually undergo IDS. Women with higher NLR values were more frequently unable to undergo debulking surgery than those with low NLR (21 vs 8 cases respectively). Although surgery was excluded from multivariate analysis, it may have still affected univariate analysis of NLR with PFS and OS. Finally, our study is based mainly on high grade ovarian cancer patients that could not be upfront debulked. Some studies support that the prognostic value of NLR may be greater in low or intermediate grade disease [40].

## Conclusions

In conclusion, NLR could serve as a useful predictor of OS but not PFS in ovarian cancer patients receiving neoadjuvant treatment. High NLR defined as over 3 may indicate patients with worse prognosis. CRS score should be implemented in everyday clinical practice as a binary prognostication system to stratify ovarian patients at high risk for relapse. Both these biomarkers require further prospective validation, while their combination in normograms along with other common prognostic factors could improve stratification of ovarian cancer patients undergoing NACT. This could be a useful tool to guide future clinical trial design and treatment personalization.

## Data Availability

Data presented in our study can be found in the patients’ archives that are safely stored in our Institution. The datasets generated during the current study are available from the corresponding author upon request.
